# Fabrication of Biocompatible Potassium Sodium Niobate Piezoelectric Ceramic as an Electroactive Implant

**DOI:** 10.3390/ma10040345

**Published:** 2017-03-26

**Authors:** Wei Chen, Zunxiong Yu, Jinshan Pang, Peng Yu, Guoxin Tan, Chengyun Ning

**Affiliations:** 1 School of Materials Science and Engineering, South China University of Technology, Guangzhou 510641, China; chenweiofcw@163.com (W.C.); conan424683@live.com (P.Y.); 2 Center for Human Tissues and Organs Degeneration, Shenzhen Institute of Advanced Technology, Chinese Academy of Science, Shenzhen 518055, China; zunxiong.yu@gmail.com; 3 Institute of Chemical Engineering and Light Industry, Guangdong University of Technology, Guangzhou 510006, China; tanguoxin@126.com

**Keywords:** potassium sodium niobate, electroactive, piezoelectric, biological, implant

## Abstract

The discovery of piezoelectricity in natural bone has attracted extensive research in emulating biological electricity for various tissue regeneration. Here, we carried out experiments to build biocompatible potassium sodium niobate (KNN) ceramics. Then, influence substrate surface charges on bovine serum albumin (BSA) protein adsorption and cell proliferation on KNN ceramics surfaces was investigated. KNN ceramics with piezoelectric constant of ~93 pC/N and relative density of ~93% were fabricated. The adsorption of protein on the positive surfaces (Ps) and negative surfaces (Ns) of KNN ceramics with piezoelectric constant of ~93 pC/N showed greater protein adsorption capacity than that on non-polarized surfaces (NPs). Biocompatibility of KNN ceramics was verified through cell culturing and live/dead cell staining of MC3T3. The cells experiment showed enhanced cell growth on the positive surfaces (Ps) and negative surfaces (Ns) compared to non-polarized surfaces (NPs). These results revealed that KNN ceramics had great potential to be used to understand the effect of surface potential on cells processes and would benefit future research in designing piezoelectric materials for tissue regeneration.

## 1. Introduction

Recently, research interest has grown in exploiting electroactive bioceramic for hard tissue implantation. Electroactive bioceramic may induce improved biological responses due to the existence of the surface electric potential in bone remodeling cycle and the piezoelectricity of bone [1,2,3,4]. The electric potentials, which occur in bones under mechanical loading, are explained partially in terms of the piezoelectric properties of the collagen [5,6,7,8,9]. This inspires the use of an electro-active implant to improve healing and adaptation of the surrounding tissue, instead of external galvanism. Piezoelectric bioceramic could be an excellent alternative to hard tissue repair for its unique electroactivity [10,11,12,13].

Electroactive bioceramic, developed for implant, includes polarized hydroxyapatite (HA) [14] and piezoelectric bioceramic in general. Polarized HA works like a capacitor that charged by applying a high level electric field (kV/mm) at elevated temperature (>200 °C) [15,16,17]. Polarized HA has, in some cases, a positive effect on biological responses, as several studies have reported that the polarized HA exhibited favorable bio-mineralization property and protein adsorption behavior [15,18,19,20,21]. However, its non-piezoelectricity and extremely low depolarization, current density below 200 °C [15] limited its application in environment in vivo. The above mentioned drawbacks can be avoided by using piezoelectric ceramic. Park et al. implanted piezoelectric barium titanate (BT) samples into canine femora and found that the longer the samples were implanted [22], the larger the voltage was produced, contributing to an increase in osseointegration. In a further study, Feng et al. found that bone growth aligned with the direction of poling using an implant of hydroxyapatite-barium titanate (HABT) composite [23]. Though it possesses passable bio-response according to the research, BT piezoelectric ceramic is difficult to apply because of its poor stability of temperature, and the cytotoxicity of barium and titanium ions cannot be ignored. At present, for potassium sodium niobate, researchers are more inclined to improving the piezoelectric properties by doping antimony (Sb), tantalum (Ta), bismuth (Bi), and other elements [24,25,26,27] with a view to replace lead zirconate titanate in industrial applications. Such doped potassium sodium niobate (KNN)-based piezoelectric ceramics have little potential in the field of implant materials due to the toxicity of the dopant elements, despite the high piezoelectric properties. Non-doped KNN has great potential in biomedical materials because of its good biocompatibility, good temperature stability (high Curie temperature), and much larger piezoelectric constant than natural bone. Thus, KNN piezoelectric ceramic could be competently used as a bone implant and the fabrication process should be investigated for biomedical application. KNN ceramic is usually fabricated by solid reaction route, which generally consists of ball milling and high temperature reaction process. Numerous fundamental studies on KNN mainly focused on the sintering temperature, which sensitively affects the physical and piezoelectric properties of ceramic for the composition segregation caused by the volatility of potassium and sodium at high temperature [28,29,30,31]. However, investigations, concerning about the effect of KNN powder fabrication that is prerequisite for controlling the physical and piezoelectric properties of ceramic, were insufficient [28,32,33,34,35].

The purpose of this paper was to fabricate an electroactive KNN piezoelectric ceramic by exploring milling time and calcination temperature and to investigate the influence of substrate surface charges on protein adsorption and cell proliferation on polarized surface compared to non-polarized surface (NPs) of KNN ceramic. In this study, we found that KNN powder fabrication was essential for controlling the piezoelectric and physical properties of KNN ceramic. Protein adsorption test with bovine serum albumin (BSA) as a normal protein model was exploited and the results revealed that both positive and negative polarized surfaces were more favorable to protein adsorption than the non-polarized samples. We also performed cells viability staining and cells proliferation experiments, the results of which revealed that KNN ceramics had the potential to be used to understand the role of surface potential on cell processes and great potential as an electroactive material in hard tissue regeneration.

## 2. Results and Discussion

**Figure 1:**
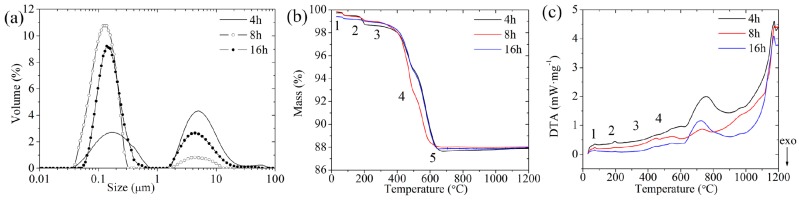
Particle size distribution, thermal gravimetric (TG), and differential thermal analysis (DTA) curves of the mixtures ball-milled for different time. (**a**) Particle size distribution of the mixtures ball-milled for 4, 8 and 16 h, respectively; (**b**) TG and (**c**) DTA curves of the mixtures ball-milled for 4, 8 and 16 h. The insert of 1,2,3,4 and 5 in Figure 1b show weight loss peaks associated with endothermic peaks of inserted 1,2,3 and 4 in Figure 1c.

**Figure 2:**
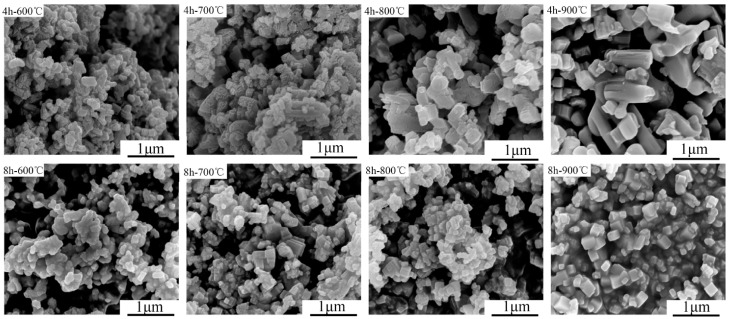
Scanning electron microscopy (SEM) images of mixture powder calcined at different temperature from 600 to 900 °C after ball milled for 4 and 8 h, respectively. When ball-milled for 8 h under the same temperature, particle size was smaller and more uniform.

Figure 1a shows the particle size distribution of the mixtures after ball milling for 4, 8 and 16 h and bimodal distribution was observed. For 4 h, in the main peak, the particle size distribution of mixtures powder ranged from 0.04 to 0.8 μm, when ball milling time was extended to 8 h, the particle size was reduced to between 0.03 and 0.3 μm. However, the size of the particles becomes larger, between 0.05 and 0.7 μm, when ball milling time was prolonged to 16 h. The above results could be explained that insufficient ball milling time led to larger particle size, while excess ball milling time would produce similar result because of reuniting. As for the diameter from 2 to 20 μm, it was a bubble due to ultrasonic dispersion, which was indirectly demonstrated by scanning electron microscopy (SEM) images of the KNN particles in Figure 2. Ball milling for 8 h was sufficient according to this study, which corresponded to the particle size distribution mainly from 30 to 300 nm.

Figure 1b,c shows the results of the TG/DTA for the raw mixtures. The samples experienced a mass loss between 11.30% and 11.86% upon heating to 700 °C. According to thermal gravimetric (TG) and differential thermal analysis (DTA) curves of Figure 1b,c, four weight loss peaks can be observed on the TG curve (Figure 1b, at 82, 185, 362, and 491 °C) associated with endothermic peaks in Figure 1c. The first weight loss occurring at 82 °C is attributed to the removal of absorbed moisture water. The weight loss peak at 185 °C is relevant to simultaneous losses of H_2_O and CO_2_ [36], which comes from the decomposition of AHCO_3_ to A_2_CO_3_ (A is K^+^ or Na^+^) [37]. The main weight losses occurred in a narrow temperature range between 400 and 600 °C, corresponding to the CO_2_ losses. The region of about 500–680 °C is due to the formation of the perovskite structure with a maximum mass loss at 650 °C. It was also observed from the TG curves that the appropriate calcination temperature is about 700 °C.

**Figure 3:**
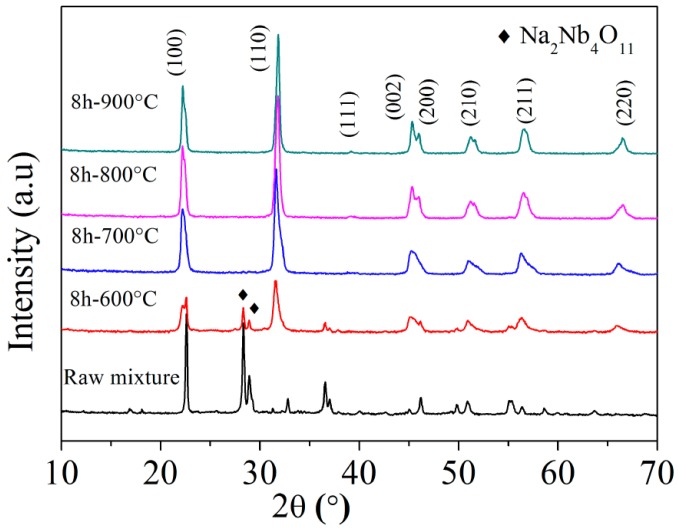
X-ray diffraction (XRD) patterns of raw material and mixture powder calcined at different temperature from 600 to 900 °C after ball milling for 8 h.

**Figure 4:**
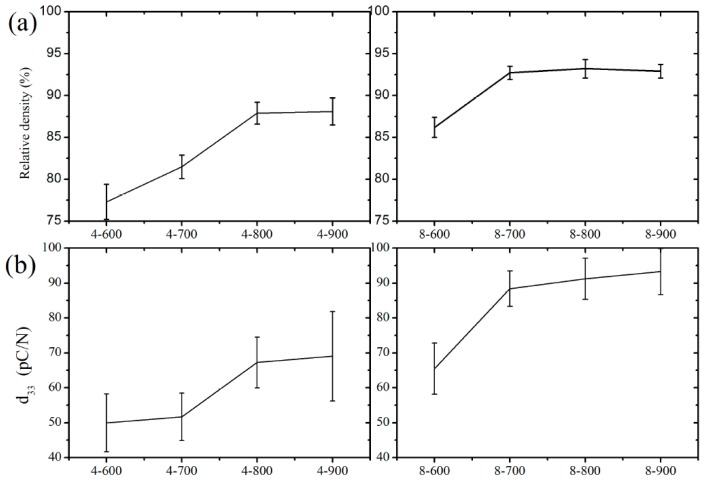
Relative density and piezoelectric constant (d_33_) of the polarized ceramic prepared from corresponding potassium sodium niobate (KNN) powder. The relationship between relative density (**a**); piezoelectric constant (d_33_) (**b**) and calcination temperature (from 600 to 900 °C) after ball-milled for 4 and 8 h, respectively.

Figure 2 showed the micro-morphology of mixture powder calcined at 600 to 900 °C after ball milled for 4 and 8 h. Despite its smaller particle size, a considerable part of impurities were still observed in the mixtures calcined at 600 °C from the result of Figure 3. With temperature increasing from 700 to 900 °C, the particle changed from random to regular. Ball-milled for 4 h and calcined at 600, 700 and 800 °C, the powder mixtures were almost random, while regular shape particle appeared at 900 °C. It could be explained by that the higher the calcination temperature, the bigger the KNN crystal grew for the 2 h compress time in this study. However, the enlarged particle was adverse to densification of the sintering process [30]. When ball-milled for 8 h and under the same temperature, the results showed evident uniformity in the particle size of powder mixtures. It could only be concluded that the mixtures milled for 8 h were advantageous to control the particle size of calcined powder. However, it is arbitrary to decide which was optimal. The appropriate fabrication process including milling time or calcination temperature was decided by the final piezoelectric and physical properties of the ceramic, as showed in Figure 4.

The crystal structure of sample powder was verified by X-ray diffraction (XRD), as shown in Figure 3. When mixtures calcined at 600 °C, it could be seen that the peak of calcined powder had weaken a lot comparing to raw material, indicating that phase transformation had occurred, but incompletely. Na_2_Nb_4_O_11_ (PDF#20-1145) was identified according to the broad peak in the 2θ from 20° to 30° [38]. However, when the temperature rose to 700 °C, it was evident that the peaks of raw materials disappeared, which was consistent with the TG-DTA result as showed in Figure 1c since complete reaction temperature of the “8 h” sample was nearly 700 °C. It was easy to see the diffraction patterns corresponded to a typical perovskite phase and the crystallographic indexing was based on the perovskite type, both for orthorhombic structure (Space Group: Amm2) and tetragonal structure (Space Group: P4mm) by using Jade 5.0 Software (MDI, Livermore, CA, USA, 2008). Besides, after calculating the ratio (I_(002)_/I_(200)_), which was about 1.496 between 1/2 and 2, the coexistence of orthorhombic and tetragonal phase was also defined [39]. Going on raising the temperature, the phase of the mixtures had changed very little, which implied stabilization coexistence between a tetragonal symmetry (T) and an orthorhombic symmetry (O) as a result of the effect of polymorphic phase boundary (PPB) in the perovskite [40]. As can be seen from Table 1, the calculated lattice parameters of both tetragonal symmetry (T) and orthorhombic symmetry (O) tended to be stable from 700 to 900 °C. It meant complete crystal structure were obtained at 700 °C.

As shown in Figure 4, the obtained relative density and piezoelectric constant of KNN ball-milled for 8 h were generally higher than that for 4 h. Piezoelectric constant (d_33_) of KNN disk samples corresponding to the calcined powder were exhibited in Figure 4b. The obtained d_33_ constant of KNN ball-milled for 8 h and calcined at 700 °C reached about 93 pC/N via regulating the powder preparation before sintering. Under the condition of ball-milled for 4 h, the obvious improvement of the relative density and piezoelectric constant (d_33_) occurred from 700 to 800 °C (left in Figure 4a,b), while under the condition of ball-milled for 8 h, the mutation happened from 600 to 700 °C (right in Figure 4a,b). The results could be concluded that the KNN grains were not formed effectively when the ball milling time was not enough and the particle size was too large, so the mixture powder needed to be melted at a higher temperature to form KNN grains. When the milling time was 8 h, the particle size is relatively uniform and fine, it was possible to react at 700 °C, which was consistent with the results of particle size distribution and the TG-DTA results of Figure 1a,c. According to Figure 2, the XRD results showed that three kinds of mixtures were supposed to react incompletely at 600 °C. Though the formation of perovskite structure was observed, it contained other phase of Na_2_Nb_4_O_11_ (PDF#20-1145) [38], leading to a low relative density of the ceramic and a decrease of the corresponding piezoelectric constant. Compared with piezoelectric ceramics used in industrial applications, 93 pC/N is small, but it is large enough for application in the piezoelectric bone implants [8]. These results indicated that the calcined temperature should be 700 °C at least and the ball milling time of the mixtures should be up to 8 h to keep capable piezoelectric properties.

**Figure 5:**
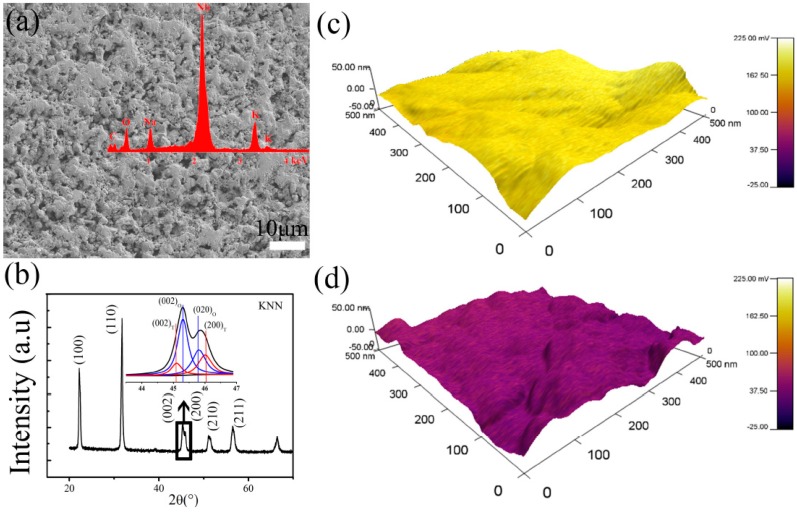

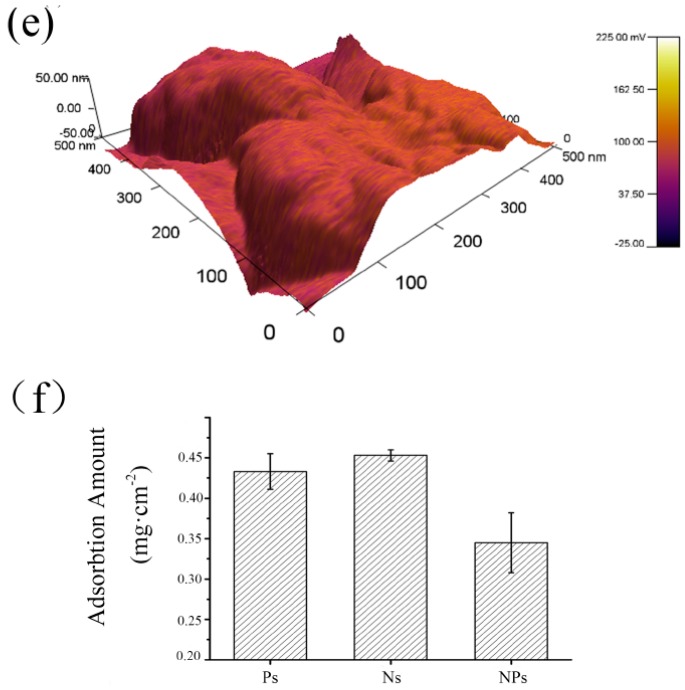
(**a**) SEM image of KNN ceramic with energy-dispersive X-ray spectroscopy (EDS) results inserted; (**b**) XRD pattern of the KNN ceramic including the insert of detail in the 2θ from 44.2° to 47°, which were fitted to the sum of four peaks indexed as two tetragonal peaks (in red) plus two orthorhombic peaks (in blue) of the perovskite phase; the surface potential by scanning Kelvin probe microscopy (SKPM) and protein adsorption behavior on three different KNN ceramic surfaces: (**c**) on positive surface (Ps); (**d**) on negative surface (Ns) and (**e**) on non-polarized surface (NPs); (**f**) the results of bovine serum albumin (BSA) protein adsorption.

KNN ceramics with piezoelectric constant of 93 pC/N prepared by sintering the die-pressed 8 h-700 °C samples in air at 1050 °C for 2 h, were chosen for SEM observation, phase inspection, surface potential study, and BSA protein adsorption testing. It showed the surface microstructure and element composition seen in Figure 5a. There were small pores between the grains, which are favorable for the contact of the material with the surrounding body fluids [41]. The XRD pattern in Figure 5b revealed that KNN ceramic was in perovskite phase with orthorhombic-tetragonal phase coexistences, and the relative volume fractions (%) of the T (V_T_) and O (V_O_) of the two-phase coexistence in this system were calculated as 39 ± 3 and 61 ± 5, respectively, from the following equations [25,42]:

V_T_ = [I_(002)_T__ + I_(200)_T__]/[I_(002)_T__ + I_(200)_T__ + I_(022)_O__ + I_(200)_O__], (1)

V_O_ = [I_(022)_O__ + I_(200)_O__]/[I_(002)_T__ + I_(200)_T__ + I_(022)_O__ + I_(200)_O__], (2)

where I_(002)_T__, I_(200)_T__, I_(022)_O__ and I_(200)_O__ are the integrated intensities of the tetragonal (002) and (200) peaks, orthorhombic (022) and (200) peaks.

As can be seen in Figure 5c–e, the surface potential of Ps, Ns, and NPs were quite different. According to the measurement and calculation by collecting the data from three randomly selected areas (500 nm × 500 nm) on KNN ceramics, the average value of surface potential were 171 ± 9, 49 ± 5, and 90 ± 8 mV in air, respectively. The potential of three KNN surfaces increased from negative surface (Ns), non-polarized surface (NPs) to positive surface (Ps) successively.

**Figure 6:**
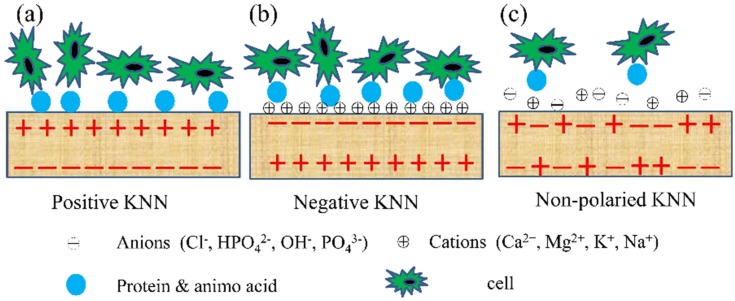
Schematic drawing of the interaction among three different surfaces of KNN and the environmental species. (**a**) On the positive KNN, anions and negatively charged proteins are actively adsorbed, forming a protein layer; (**b**) On the negative KNN, cations, particularly Ca^2+^, are selectively adsorbed ,leading to the presence of positively charged ion layer, which in turn promote prot ein adsorption and proliferation cells on this surface; (**c**) On non-polarized KNN surface, inorganic ions, amino acids and proteins float and attach to non-polarized KNN.

The amount of BSA adsorption on positive surface (Ps) and negative surface (Ns) were higher than that on non-polarized surface (NPs) in Figure 5f. In general, each protein or amino acid has an isoelectric point (the pH of solution) where they exhibited an electrically neutral state. When the pH of solution was higher or lower than isoelectric point of a protein or amino acid, it displayed negative or positive in solution, respectively. The isoelectric point of BSA protein is 4.7, so BSA protein is negative when dissolved in phosphate buffer solution (PBS) (pH = 7.4) [15]. In this work, both positive and negative surfaces exhibited enhanced protein adsorption than non-polarized surfaces, the result of which could be explained in term of ion crowding in the electric double layer [43,44,45], whereby the crowding of counterions dominates screening. When KNN were immersed into BSA solution, negative BSA proteins were selectively adsorbed on positive KNN surface. While on negative KNN surface, cations (K^+^, Na^+^) were closely attracted, leading to the presence of positively charged ion layer, then absorbed negative BSA. In addition, it was also reported that proteins would experience conformational changes when absorbed onto solid surface [46,47], which affected the amount of adsorption. Although understanding about the conformational changes is not enough, it has been demonstrated that surface charge has a great impact on this process [48,49]. The adsorption mechanism can refer to Figure 6, a schematic illustration for cell proliferation. In this study, BSA proteins were more attracted to the charged KNN surfaces just like anion in the electric double layer compared to uncharged KNN surfaces, as our results showed.

**Figure 7:**
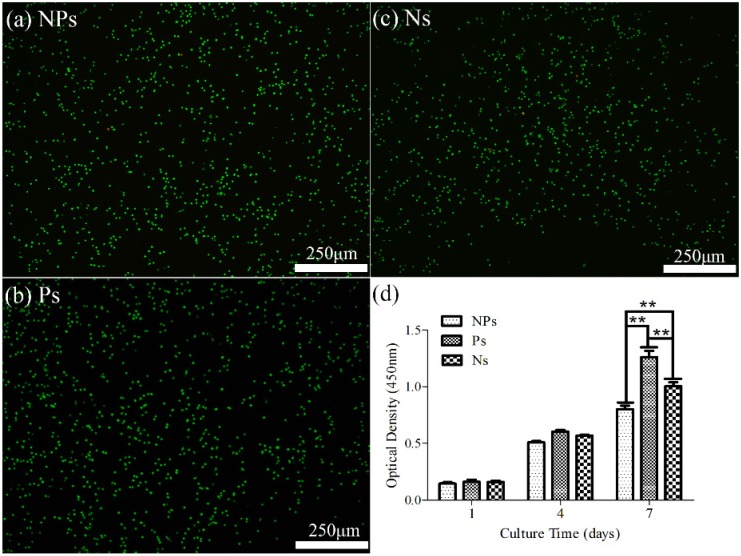
Cell viability on the surfaces of KNN piezoelectric ceramics: Calcein AM to detect live cells, propidium iodide to detect dead cells. (**a**) non-polarized surfaces (NPs); (**b**) positive surfaces (Ps) and (**c**) negative surfaces (Ns); (**d**) Cell proliferation on NPs, Ps and Ns was determined by CCK-8 assay on days 1, 4 and 7 days. The ** indicated significant difference (*p* < 0.01).

To assess cell viability, MC3T3-E1 osteoblasts were seeded on KNN ceramics with piezoelectric constant of 93 pC/N and after 24 h were stained with calcein for live (in green) and with propidium iodide for dead (in red). The fact that only a few dead cells (stained in red) were observed showed that the synthetic KNN ceramics did not cause damage to cells, namely they were non-toxic, whether they were polarized or not. To further study their biocompatibility, cells proliferation experiment was performed. The study on the proliferation (Figure 7d) of MC3T3-E1 osteoblasts using cell counting kit 8 (CCK-8) assays revealed that Ps and Ns ceramics promoted cell growth more significantly than NPs samples for approximately seven days. Although BSA was not directly related to cell growth, the mechanism of BSA protein adsorption can also be applied to the cell proliferation. The culture medium mimicking the physiological environment contained different chemicals that had the same opportunity to contact and to be adsorbed on the surface of KNN ceramic. However, the charged groups in the medium can be repulsed or attracted by the surface charge of KNN. An explanation was made in term of ion crowding in the electric double layer [43,44,45], whereby the crowding of counterions dominates screening. When KNN were immersed into cell culture medium, since most proteins are negatively charged under physiological conditions, as a result of an isoelectric point less than 6 [50,51], negative protein were selectively adsorbed on positive KNN surface, forming a protein layer that contributes to cell adhesion [52] (Figure 6a). While on negative KNN surface, cations including Ca^2+^ counter-ions, which is considered to promote osteoblast cell adhesion by attracting negatively charged proteins from cell medium [16] and by supplying Ca^2+^ to binding sites in transmembrane proteins responsible for cell adhesion and proliferation [53], were closely attracted, contributing to the adhesion and proliferation of cells on this surface (Figure 6b). On non-polarized KNN surface, inorganic ions, amino acids and proteins float and attach to non-polarized KNN assuming that the non-polarized surface was neutral, compared to the negative surface and the positive surface (Figure 6c). In addition, considering that cell size (10–20 μm) is bigger than the positive or negative regions (several hundred nanometers) [54] in non-polarized KNN, the absorption due to electrostatic effects was more difficulty. The biocompatible KNN piezoelectric ceramics obtained in this study can be an ideal candidate model to investigate the interaction between surface charge and multi-potential cells such as stem cells in the future. The results from cell viability and proliferation assay (shown in Figure 7a–d) clearly showed the great potential of polarized KNN ceramics as a new biomaterial with electroactivity for bone tissue regeneration.

## 3. Materials and Methods

### 3.1. Samples Preparation

The KNN piezoelectric ceramic powder was synthesized by solid state reaction using the raw materials of sodium carbonate (Na_2_CO_3_, 99.8%, Sinopharm Chemical Reagent Co. Ltd., Shanghai, China), potassium carbonate (K_2_CO3, 99.0%, Sinopharm Chemical Reagent Co. Ltd., Shanghai, China), and niobium oxide (Nb_2_O_5_, 99.9%, Shanghai Aladdin, Shanghai, China) at the mole ratio of r(K:Na:Nb) = 0.5:0.5:1. After drying at 120 °C for 5 h, the raw materials were weighed and added to a Teflon bottle along with agate balls of 6 mm and 10 mm in diameter. The ratio of powder mixtures to balls to liquid was approximately 1:4:4 by mass. Then the mixtures were ball-milled by planetary using ethanol as medium for 4, 8 and 16 h, respectively. The dried mixtures were calcined in an alumina crucible at different temperature of 600, 700, 800, and 900 °C for 2 h, respectively. Adding 8 wt % of polyvinyl alcohol as binder, the calcined powder mixtures were then die-pressed into discs (diameter 10 mm, thickness 3 mm) under 150 MPa without second ball milling and sintered in air at 1050 °C for 2 h in a loosely-covered Al_2_O_3_ crucible.

### 3.2. Poling and Protein Adsorption

After gold electrode was sprayed on both surfaces, KNN disks were poled at the electric field of 2.5 kV/mm under 100 °C in silicone oil for 15 min using piezoelectric polarization device (HYJH-3-4, Huiyuan Automation Equipment Co., Ltd., Xianyang, China). After polishing and ultrasonic cleaning, the disks were immersed into the phosphate buffer solution (PBS, 1×, Gibco, Carlsbad, CA, USA) for 24 h and then wiped up the residual liquid on both surfaces. After drying, differently polarized KNN disks with a diameter of 10 mm were immersed 1 mL of protein solution (1 mg/mL) and incubated at 37 °C for 10 h with the opposite surface sticking to the bottom of 24-well plate. Then the protein solution was removed, followed by transferring the disks to a new plate. The amount of the absorbed protein on KNN ceramics were determined by bicinchoninic acid (BCA) assay. The protein concentration of remnant solution was tested 562 nm by microplate reader (Thermo Scientific, Waltham, MA, USA). The experiment was repeated at least three times and a mean value was calculated.

### 3.3. Live/Dead Cell Staining and Proliferation Assay

Positive polarized, negative polarized, and non-polarized KNN ceramics with piezoelectric constant of 93 pC/N were sterilized by autoclaving at 120 °C for 30 min and then placed in 48-well plates. MC3T3-E1 osteoblasts were cultured in α-modified minimum essential medium (α-MEM) supplemented with 10% fetal bovine serum (FBS) in a humidified incubator at 37 °C and 5% CO_2_. MC3T3-E1 osteoblasts were seed on the specimens at 2 × 10^4^ cells/mL for live/dead cell staining for 24 h and cell proliferation for 1, 4 and 7 days, respectively. The medium was changed every two days for the duration of the experiment. Dulbecco’s Phosphate Buffered Saline (DPBS) solutions supplemented with 2 mL (1 mg/mL) calcein-AM and 2 mL (1 mg/mL) propidium iodide was used for live and dead cells staining, respectively. After incubation for 40 min at 37 °C and 5% CO_2_, the samples were washed with DPBS and were imaged using inverted fluorescence microscope (Shinjuku, Tokyo, Japan). Cell Counting Kit-8 (CCK-8) assay was used to evaluate cell proliferation at 1, 4, and 7 days.

### 3.4. Characterizations

Particle size distribution of ball-milled mixture was determined by laser particle analyzer (LPA) (Mastersizer 2000, Malvern, England) using dehydrated ethanol as the solvent. Thermal gravimetric (TG) and differential thermal analysis (DTA) (NETZSCH-STA449C, Bavaria, Germany) were used for characterizing the thermo properties of the mixtures, with a heating rate of 5 K/min from room temperature to 1200 °C. The compositions and morphology of calcined powder were examined by X-Ray Diffraction (XRD, X’Pert Pro, PANalytical, Amsterdam, the Netherlands) and scanning electron microscopy (Evo 50, Zeiss, Oberkochen, Germany), respectively. Jade 5.0 program was used to analyze X-ray diffraction data. Density of the ceramic was measured using Archimedes principle. d_33_ meter (YE2730A, YuTian Technology Co., Ltd., Wuxi, China) was used for measuring piezoelectric constant of polarized ceramic disks. The surface potentials of Ps, Ns, and NPs of KNN were determined by scanning Kelvin probe microscopy (SKPM) (Multimode 8, Bruker, Blaireka, USA). Bicinchoninic acid (BCA) assay (Cwbio, Shanghai, China) was used to determine the protein adsorption amount. The proliferation measurement was quantified using a Cell Counting Kit-8 (CCK-8) assay (Shanghai Beyotime Institute of Biotechnology, Shanghai, China) and Prism 5.0 software (GraphPad software Inc., La Jolla, CA, USA, 2012) was applied to deal with the data. Significance levels were set at ** < 0.01.

## 4. Conclusions

Biocompatible KNN ceramic with piezoelectric constant of ~93 pC/N was prepared through adjusting ball-milling time and calcination temperature and was considered to be a promising electro-active biomaterial for tissue implant. Evidence of TG-DTA curves and XRD patterns revealed that the complete solid reaction product with particle size of 0.03–0.3 μm could be obtained by ball milling for 8 h and then calcined at 700 °C for 2 h. The potentials of positive surfaces (Ps), negative surfaces (Ns) and non-polarized surfaces of KNN were 171 ± 9, 49 ± 5, and 90 ± 8 mV as evaluated, respectively. Both positive and negative surfaces exhibited enhanced protein adsorption and cell proliferation over non-polarized surfaces, suggesting that role of surface potential on protein adsorption and cells proliferation depended primarily on the presence of charge. KNN ceramics can be used to understand the role of surface potential on cells processes and great potential as an electroactive material in hard tissue regeneration.

## Figures and Tables

**Table 1:** Structural parameters of the powder determined by Jade 5.0 software at different calcination temperature.

Temperature (°C)	Lattice Parameters of the O Phase			Lattice Parameters of the T Phase	
	*a* (Å)	*b* (Å)	*c* (Å)	*a* = *b* (Å)	*c* (Å)
700	3.9722	5.6297	5.7667	3.9969	3.9889
800	3.9743	5.6329	5.7696	3.9874	4.0228
900	3.9774	5.6369	5.7685	3.9904	4.0054
